# Unveiling Nature’s Architecture: Geometric Morphometrics as an Analytical Tool in Plant Biology

**DOI:** 10.3390/plants14050808

**Published:** 2025-03-05

**Authors:** Federica Spani, Vittoria Locato, Laura De Gara

**Affiliations:** 1 Department of Science and Technology for Sustainable Development and One Health, Research Unit of Food and Nutrition Sciences, Università Campus Bio-Medico di Roma, Via Alvaro del Portillo, 21, 00128 Roma, Italy; v.locato@unicampus.it (V.L.); l.degara@unicampus.it (L.D.G.); 2 National Biodiversity Future Center, NBFC, 90133 Palermo, Italy

**Keywords:** plant science, shape variation, size variation, PRISMA 2020, landmark-based methods, plant organs

## Abstract

Geometric morphometrics (GMM) is an advanced morphometric method enabling quantitative analysis of shape and size variations in biological structures. Through high-resolution imaging and mathematical algorithms, GMM provides valuable insights into taxonomy, ecology, and evolution, making it increasingly relevant in plant science. This review synthesizes the existing literature and explores methodological details, research questions, and future directions, establishing a strong foundation for further study in plant biology. Following PRISMA 2020 guidelines, a rigorous literature search finally identified 83 studies for review. The review organized data on plant species, organs studied, GMM objectives, and methodological aspects, such as imaging and landmark positioning. Leaf and flower structures emerged as the most frequently analyzed organs, primarily in studies of shape variations. This review assesses the use of GMM in plant sciences, identifying knowledge gaps and inconsistencies, and suggesting areas for future research. By highlighting unaddressed topics and emerging trends, the review aims to guide researchers towards methodological challenges and innovations necessary for advancing the field.

## 1. Introduction

Morphometrics is the quantitative description, analysis, and interpretation of shape and its variations [1]. In biology, it represents a topic of growing interest for studying both animal and plant anatomical structures. In the field of animal biology, morphometrics allows researchers to investigate the influence of environmental variables on ontogenesis and the resulting shapes of organs and tissues, with implications for species evolution and ecology [2,3,4,5]. Similarly, in plant biology, this discipline enables the analysis of how environmental factors such as light, temperature, and nutrient availability influence the morphology of leaves, flowers, fruits, and other structures, providing tools to understand functional and ecological adaptations in the plant kingdom [6,7].

### 1.1. Traditional Morphometrics

Traditional morphometrics considers lengths, widths, masses, angles, ratios, and areas for shape analysis [8]. However, this approach has several limitations, chief among them being the high autocorrelation of measurements and the challenges in identifying the causes underlying shape variation within the examined structure [9]. In particular, traditional morphometric variables are often highly correlated, making it difficult to determine whether observed differences reflect true biological variation or are merely artifacts of measurement redundancy. Furthermore, this method relies heavily on linear distances and ratios, which may fail to capture complex shape differences effectively, especially in highly variable structures like leaves, flowers, and seeds. Another critical drawback is the lack of spatial information: traditional morphometrics does not preserve the relative positions of measured points, making it unsuitable for studying morphological patterns that depend on geometric relationships. These and other limitations have driven a revolution in morphometric investigation techniques over the past decades, starting in the early 1980s [9,10].

### 1.2. Geometric Morphometrics

Strauss and Bookstein [11] recognized the limitations of traditional morphometric approaches, emphasizing how they provide very limited and redundant information about the shape of a given structure. They highlighted the need to consider the geometric relationships among the variables under study and stressed that their selection should be based on ontogenetic models [11]. As the name suggests, geometric morphometrics (GMM) focuses on the geometric relationships of a set of homologous points, referred to as landmarks. Numerous methods have been developed that account for (and exclude from the analysis) non-shape variations, such as size, orientation, and translation, to minimize their influence on shape analysis and provide more reliable results compared to traditional morphometrics [1,12].

The use of geometric morphometrics in biological sciences has increased significantly over recent decades. This is evidenced by the growing prominence of the keywords “geometric” and “morphometrics” in Google Scholar, with approximately 50 results in 1998, 225 in 2007 [13], and around 76,000 as of 2024. The applications are diverse, ranging from studies on intraspecific and interspecific variability, and phylogeography [14,15], to investigations into the types and degrees of asymmetry in biological structures—likely indicators of intrinsic or extrinsic disruptions in the ontogenetic process. Such disruptions can lead naturally symmetrical structures to develop varying degrees of asymmetry [16]. According to this paradigm, several authors have hypothesized that fluctuating asymmetry could serve as a proxy for environmental or genetic stress [16,17]. Unlike other known forms of asymmetry, which are considered adaptive—such as directional asymmetry and antisymmetry—fluctuating asymmetry, whether at the population level (in its original conception) or at the individual level, does not confer adaptive advantages. Geometric morphometrics is particularly well-suited for such applications, as it can quantify even minimal variations and differences in shape with greater accuracy than traditional morphometric approaches [18,19].

Various tools are available to analyze geometric variables in two and three dimensions. All methods use Cartesian coordinates of comparable features on the examined structure—landmarks, curves, and contours—as their variables [13]. Most methods transform points, curves, and contours into shape coordinates, derived variables that can be analyzed using classical multivariate statistical tools. Other approaches convert the data into a distance matrix between the variables of interest, such as the Euclidean Distance Matrix Analysis (EDMA), which aligns more closely with traditional methods [13].

The most common geometric morphometric tools for shape analysis based on landmarks use Procrustes superimposition [12,20,21,22]. This method rotates, translates, and scales the structures under study (based on the positions of landmarks) to achieve homologous orientation and size, minimizing the mean squared differences between homologous landmarks. Removing differences in orientation, position, and size allows for the analysis of pure shape [12].

There are also various methods for analyzing curves and contours. These are often studied using semi-landmarks, points placed at defined intervals along curves and between two landmarks [23,24]. Semi-landmarks are also superimposed using the Procrustes method, similar to regular landmarks, though they are considered “deficient” since their placement does not rely on the identification of ontogenetically conserved features on the examined structure [25]. Another method for contour analysis is Fourier analysis, one of the oldest morphometric methodologies. This method uses sine and cosine harmonic functions to describe the position of contour coordinates [26]. Eigenshape analysis is a third method for contour and curve analysis [27,28,29], which converts coordinates along a curve or contour into a function φ, essentially a list of angles from one point to the next in the series under study. Despite the variety of methods available, contour analysis is often criticized for the previously mentioned issues—namely, that the individual coordinate points are not biologically homologous [30].

Once shape coordinates are derived—representing a reduced set of variables derived from landmarks that collectively describe shape variation—any type of multivariate statistical analysis can be applied using the previously mentioned methods [25,31,32].

The application of GMM in plant science is vast and varied, spanning across diverse disciplines such as taxonomy, systematics, ecology, plant physiology, paleobotany, and evolutionary and developmental biology [33,34,35,36]. Christodoulou and colleagues [37] revised for the first time the combination of GMM with statistical/machine learning analyses for plant classification, emphasizing the importance of evaluating performance using standardized metrics like accuracy, sensitivity, and specificity, as well as the practicality of GMM approaches for non-destructive plant sample identification due to advancements in high-resolution imaging and desktop computing power. From characterizing leaf shapes for species identification to unraveling the morphological consequences of environmental stressors on plant populations [38], GMM offers invaluable insights into the functional significance, adaptive strategies, and evolutionary trajectories of plants [39]. Starting from this, this review aims to provide an updated exploration of GMM in plant science, covering its principles, methodologies, applications, and implications. It deepens methodological intricacies, challenges, and future directions, addressing topics such as image acquisition, landmark placement, statistical analysis, and interpretation. Furthermore, it discusses the diverse research questions addressed by geometric morphometric analyses in plant morphology, evolution, ecology, systematics, and paleobotany, while also evaluating the strengths, limitations, and challenges associated with its application in plant biology.

## 2. PRISMA 2020 Guidelines Applied to Systematic Reviews in Plant Morphometrics

The literature search conducted for this systematic review followed the guidelines provided by Preferred Reporting Items for Systematic Reviews and Meta-Analysis (PRISMA), which was first published by Moher and colleagues [40], but then updated by Page and colleagues [41] to the new version known as PRISMA 2020.

The PRISMA 2020 method is a widely recognized guideline for conducting systematic reviews and meta-analyses of scientific literature [40,41]. This method was developed to improve the transparency and quality of reporting in these types of studies and is considered the gold standard in the field. The PRISMA 2020 method consists of a 27-item checklist that outlines the key components of a systematic review or meta-analysis. These items cover all aspects of the study, including the research question, search strategy, selection criteria, data extraction, and data analysis. By following the PRISMA 2020 checklist and adapting it, if necessary, to the objective of the review (as not all items are easily implemented in all review papers), researchers can ensure that their study is comprehensive, transparent, and reproducible.

The databases of peer-reviewed literature checked were Scopus (19 January 2024) and Web of Science (19 January 2024). Keywords searched in all fields (e.g., title, author, keywords, abstract, and text) were ‘Geometric’ AND ‘Morphometric’ AND ‘plant,’ ‘Geometric’ AND ‘Morphometric’ AND ‘phenotyping,’ and ‘Geometric’ AND ‘Morphometric’ AND ‘plant’ AND ‘phenotype.’ Selected peer-reviewed articles have been published in national and international journals, and in a time range of 16 years (2008–2024).

**Figure 1:**
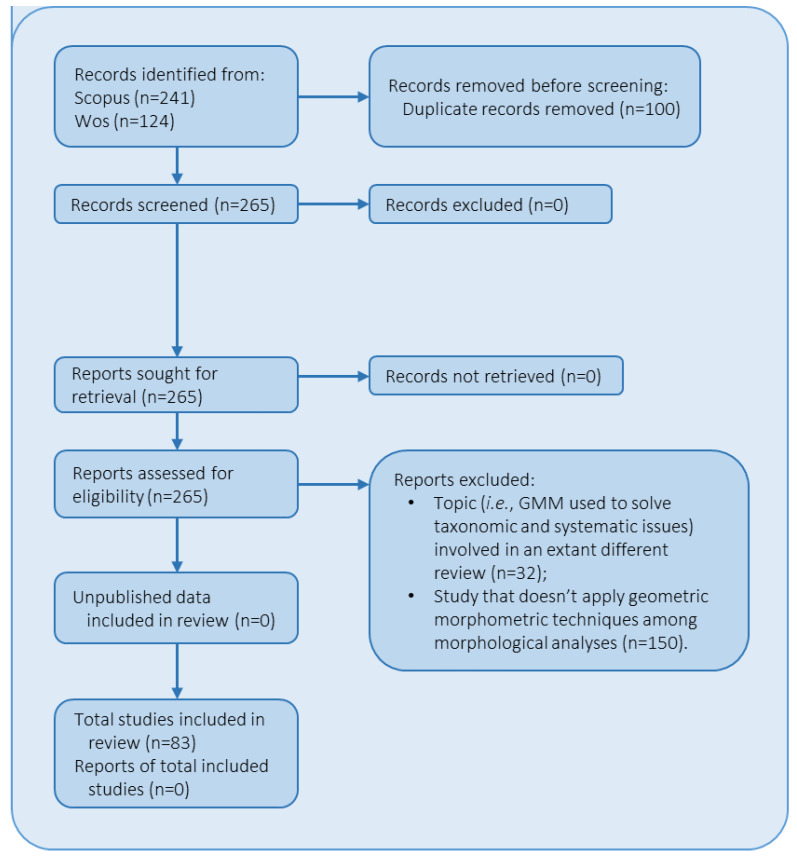
The Preferred Reporting Items for Systematic Reviews and Meta-Analyses (PRISMA) 2020 flow diagram describing the selection process of reports included in the present review. The design has been adapted from a flow diagram published by Page et al. [41].

In addition to the checklist, the PRISMA method also includes a flow diagram (Figure 1) that visually represents the flow of information through the different stages of the review process. This diagram helps researchers to track the selection and inclusion of studies in their review and can be a useful tool for identifying potential biases and gaps in the literature.

Redundant articles were removed. After the collection of full-text articles, several reports were excluded following two main exclusion criteria: (1) The study was already involved in a previously published review that focused on the use of GMM to solve taxonomic and systematic issues in plant biology (Christodoulou et al., 2020), and (2) the study did not apply GMM as tool for assessing morphological variation of plant structures.

A qualitative synthesis was conducted on selected reports focusing on the investigated morphological variation in plants using GMM. Data from each report were collected to analyze different aspects of the studies. The evaluation considered the use of 2D and 3D landmarks and manual and semi-automatic positioning in the reports. Then, synthesis categorized reports based on the types of plant sample studied and examined the distribution of objectives analyzed through GMM. This allowed for the identification of most common research fields and of those requiring further investigation.

## 3. Screening and Analysis of GMM Studies in Plant Science Research

A total of 365 articles were identified through database searching. After removing all duplicates (n = 100), 265 abstracts of selected records were screened, and each was thoroughly examined. The full text was then considered for eligibility. Based on the exclusion criteria (refer to previous section and Figure 1), 182 out of 265 were not included in the present work, leaving a final number of 83 studies included in the review (see Table S1 for the complete list of selected reports and related collected data).

The collected information pertained to both the investigated plant species and samples. In examining the material and method sections, details such as the type of imaging applied (either 2D or 3D), the total number of landmarks and semi-landmarks positioned (if available) for each plant organ, the kind of landmark positioning performed (either manual or semi-automatic), and the list of correlates were recorded. Subsequently, the specific issues analyzed through GMM were also identified for each selected work. Finally, considering all the previous information, a general topic was assigned to each report to integrate it into a broader research field, enabling a more comprehensive classification of the entire work.

The total number of reports using 2D landmarks was much higher (n = 76) than those using 3D landmarks (n = 7). Among the former, the majority used manual positioning of 2D landmarks (n = 63), while a very low number of studies employed semi-automatic methods to place 2D landmarks (n = 13). Regarding works using 3D landmarks, most of them were positioned manually (n = 5), while the remaining studies (n = 2) applied semi-automatic methods for placing 3D landmarks.

**Figure 2:**
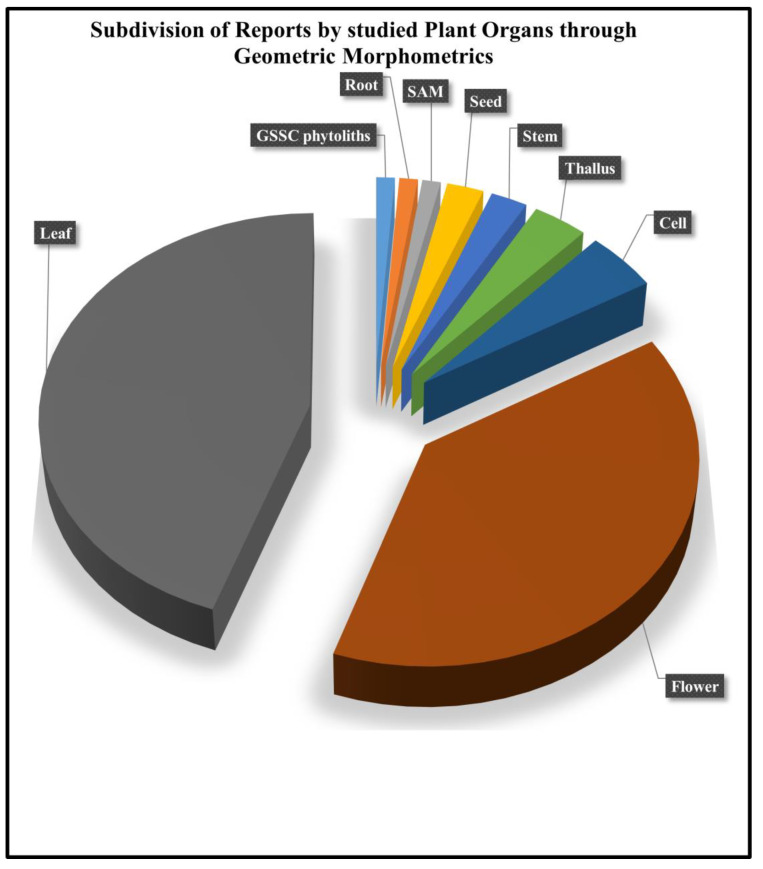
Subdivision of the reports selected for this review according to the type of plant sample studied through GMM: leaf (48.8%), flower (39.8%), cell (4.8%), thallus (3.5%), stem (2.4%), seed (2.4%), shoot apical meristem (SAM—1.2%), root (1.2%), and grass silica short cell (GSSC) phytoliths (1.2%).

Plant samples analyzed using the GMM technique, as identified through bibliographic review, included cells, flowers, grass silica short cell (GSSC) phytoliths, leaves, roots, seeds, shoot apical meristems (SAMs), stems, and thalli (Figure 2). Among these, the leaf was the most frequently studied (38 out of 83 works), followed by the flower (33 out of 83 works). Together, these two plant organs account for about 85% of the studies reviewed, while the remaining plant samples were each addressed in less than 5% of the total works.

**Figure 3:**
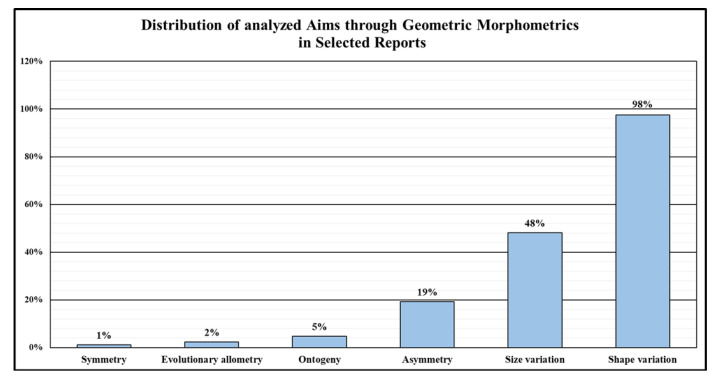
Distribution of reports (%) based on the main objective analyzed through geometric morphometrics.

When considering the subjects analyzed in the studies, GMM was chosen as the approach to primarily investigate shape variation of a plant organ (81 out of 83 works), followed by the analyses of size variation (38 out of 83 works) and asymmetry (16 out of 83 works). The remaining issues analyzed using GMM included ontogeny, evolutionary allometry, and the symmetry of specific plant samples, totaling 7 reports (Figure 3).

**Figure 4:**
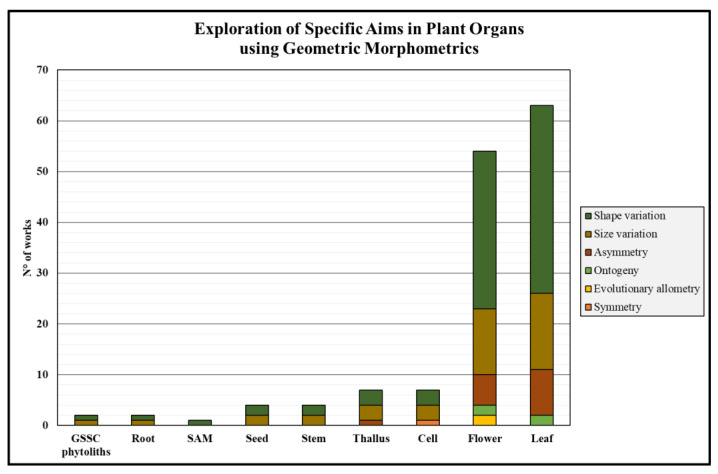
Distribution of specific issues investigated through geometric morphometrics for each plant organ.

By integrating information on plant specimens and topics primarily investigated using GMM, it was possible to determine the specific subjects typically explored in each plant sample employing GMM (Figure 4). The analysis revealed that shape variation of the leaf was the most frequent target of GMM (34 studies), followed by the studies of size variation (15 studies), asymmetry (9 studies), and ontogeny (2 studies). Similarly, in studies involving flowers, shape variation was the most commonly studied aim through GMM (33 studies), followed by size variation (12), asymmetry (6), and ontogeny (2). Additionally, a new target, evolutionary allometry, appeared for the first time (2 studies). In studies on unicellular green algae belonging to the *Micrasterias* genus, shape (3) and size (2) variation were equally investigated using GMM, with a new focus emerging: symmetry (1). A similar trend was observed in morphological studies on the thallus of a calcareous green seaweed, where shape (3) and size (3) variation were equally examined, along with the addition of asymmetry (1). Regarding seed and stem organs, both shape (2) and size (2) variation were equally studied using GMM. The sole report retrieved on the shoot apical meristem (SAM) focused on its shape variation. Finally, studies on roots and grass silica short cell (GSSC) phytoliths equally focused on the shape (1) and size (1) variation of the investigated organs.

Ultimately, we analyzed the distribution of examined studies within plant science’s hot topics (i.e., plant-environment co-evolution; plant adaptation to different habitats; plant reshape in response to environmental changes; plant biodiversity; plant development; anthropic effect on plant morphology; genetic determinants of plant morphology). This analysis was crucial for organizing the chosen records based on their relevance to specific research fields and pinpointing areas warranting further investigation in future research endeavors. It is important to emphasize the concept that a single study could be included in different topics, given that one or more objectives may align with different research fields. Notably, one of the least represented topics in plant science was ‘Plant development,’ which appeared in only two case studies from the bibliographic research. On the other hand, the most studied topic was ’Plant–environment co-evolution,’ which was extensively covered in the literature. Considering this complex scenario, the following section will discuss the selected studies within a specific research topic division, but will also consider the possibility of multiple works being included in more than one topic.

## 4. Evaluating GMM Contributions Across Key Plant Biology Research Areas

### 4.1. Plant–Environment Co-Evolution

A different set of studies broadens the intricate evolutionary relationship between flower morphology and pollination mechanisms across various plant species. Benitez-Vieyra et al. [42] examined the influence of floral shape on pollinator preferences in orchids, revealing unexpected outcomes despite visual similarity to female wasps. Bilbao et al. [43] identified significant differences in petal shape between passerine-pollinated and different order bird-pollinated *Erythrina* species, emphasizing the utility of petal combinations in inferring pollinator types. Meanwhile, Faure et al. [44] explored correlations between corolla shape in Antillean Gesneriaceae and the bill shape of hummingbird pollinators, revealing distinct relationships between pollination specialists and generalists. Further investigations by García et al. [45] on *Nicotiana glauca* (Graham, 1828) revealed how flower shape and length vary across different pollination environments. They observed that in regions with specialized pollinators, the flowers exhibited longer, more tubular shapes, while in areas with generalist pollinators, flowers were shorter and more open. These variations demonstrate the complex relationship between plant morphology and the selective pressures exerted by different pollinator types, highlighting the role of morphological traits in facilitating efficient pollination strategies. Additionally, Gómez et al. [46,47] found a negative association between corolla-shape integration and the number of pollinators in *Erysimum* genus. Their studies showed that species with more integrated corolla shapes tended to attract fewer pollinators, suggesting that highly specialized flower shapes may be less effective in systems with a high number of pollinator types. This observation points to conflicting selection pressures, where specialized flower shapes are favored in certain environments, while more generalized forms may be better suited for broader pollinator interactions in diverse ecosystems. Budečević et al. [48] investigated the evolution of flower traits in *Iris pumila* L., focusing on various characteristics including color, size, shape, and fluctuating asymmetry (FA) in floral organs. The study specifically compared traits of flowers that were successfully pollinated to those that were not. Their findings revealed that pollinated flowers exhibited higher brightness compared to the flowers that did not succeed in attracting pollinators. This suggests that pollinator-mediated selection might favor brighter flowers, possibly because they are more visually appealing to pollinators. Additionally, the study found that pollinated flowers also had longer stems and larger floral organs, which could further enhance their visibility and attractiveness, indicating that these traits collectively contribute to pollination success. Strelin et al. [49] explored ontogenetic allometry in *Calceolaria polyrhiza* Cav., a species relying on oil-collecting bees for pollination. They found that non-allometric variation in shape contributes significantly to adaptation, especially in response to shifts in pollinator size, resulting in shape changes that occur early in flower development. These studies collectively enrich our understanding of the complex interplay between floral traits and pollinator interactions, offering insights into the evolutionary dynamics shaping plant–pollinator relationships.

The work of Strelin et al. [39] is a perfect example of how different topics can be related to each other. It aimed to investigate the adaptive radiation (topic: plant–environment co-evolution) of pollination syndromes in *Caiophora*, focusing on whether it occurred through ontogenetic scaling or involved a departure from the basal ontogenetic pattern (topic: plant development). They used geometric morphometric variables to describe the shape and size of the floral structures involved in pollination mechanisms. The study revealed that derived *Caiophora* species exhibited divergent ontogenetic patterns from basal species, suggesting significant changes in the developmental pattern of flowers during the adaptive radiation, thus rejecting the ontogenetic scaling hypothesis, which posits that evolutionary changes in size and shape occur through proportional scaling of a shared ancestral developmental trajectory [39]. The last set of selected reports included in this topic focused on different aspects of the morphological evolution of flowers. McCarthy et al. [33,34] investigated the effects of allopolyploidy on floral morphology in *Nicotiana* genus, revealing that polyploid morphological divergence from progenitor phenotypes increases over time, particularly in floral limb shape and corolla tube length. They found that most polyploids exhibit distinct or transgressive traits, evolving shorter and wider corolla tubes, potentially leading to a shift towards more generalist pollination strategies. In contrast, O’Hanlon et al. [50] explored the mimetic relationships of the orchid mantis with model flower species in Malaysia, concluding that the mantis resembles an average or generalized flower-like stimulus rather than mimicking a specific model species. Rubini Pisano et al. [36] examined phenotypic variation and hybridization in a *Mandevilla* population, discovering extensive hybridization between *M. laxa* (Ruiz and Pav.) Woodson and *M. pentlandiana* (DC.) Woodson, resulting in a hybrid swarm with transgressive phenotypes in floral volatiles and asymmetrical rates of backcrossing.

In the specific context of flower–pollination strategy co-evolution, Hsu et al. [51] utilized X-ray micro-computed tomography (micro-CT) to capture 3D structures of *Corytholoma* clade corollas, extracting 415 3D landmarks from each specimen. Evolutionary allometry in corolla shape was weak. Four morphological traits were defined, including tube curvature and dilation, which were measured using 3D landmark analysis to quantify the bending and expansion of the corolla tube. Tube curvature refers to the degree of bending along the tube’s longitudinal axis, while dilation indicates the extent of widening or expansion in the tube structure. These traits were found to be strongly linked to pollination type in the *Corytholoma* clade. Ibañez et al. [52] investigated flower morphology variation in *Jaborosa* species, finding significant differences associated with pollination modes, particularly in sagittal view, suggesting adaptation to different pollinators. Similarly, Joly et al. [53] explored corolla shape variation in Gesneriinae, indicating distinct shapes among hummingbird specialists, bat specialists, and mixed-pollination species. Finally, Wolcott et al. [54] employed micro-CT and geometric morphometric analysis to study cacao’s pollination biology, identifying the petal side door as a bottleneck for pollinator access and proposing new evidence for predicting unknown pollinators.

Concerning leaf plant organs, Gallaher et al. [55] investigated leaf shape and size variation in Poaceae across habitats and climates, revealing convergent evolution of grass leaf shape towards different optima in forest understory, margins, and open habitats, with leaf size variation correlated with habitat conditions, suggesting transitional zones between forests and grasslands played a crucial role in shaping leaf morphology and ancestral habitat. This report could be also included in the topic, plant adaptability to different habitats.

Hanušová et al. [56] investigated introgression levels in the taxonomically challenging *Diphasiastrum* genus of the Lycopodiaceae family through flow cytometry and geometric morphometric analyses on both the dorsal and ventral sides of the stem, revealing continuous patterns of stem morphological variation and genome size correlation indicative of extensive interspecific gene flow within Central Europe.

Another organ investigated was the shoot apical meristem (SAM) by Schnablová et al. [57], who were interested in the relationship between SAM shapes and various shoot traits across 110 herbaceous angiosperms. They found that SAM shapes were diverse across lineages, but exhibited strong phylogenetic conservatism, with variations in SAM shapes associated with stem thickness, leaf area, and leafiness, suggesting geometric interdependence of meristem zones gives rise to correlations among organ traits and indicating conservatism in regulatory processes underlying plant architecture. This study is also related to the plant biodiversity topic.

Moving on to different plant specimens, Gallaher et al. [58] conducted a study focusing on fossil grass silica short cell phytoliths (GSSCPs) to improve their classification accuracy and biogeographical reconstructions. By using in situ observations and confocal microscopy to generate 3D models, they analyzed GSSCP shape and size variation across different Poaceae subfamilies. Their findings revealed significant shape differences among recognized GSSCP morphotypes and provided insights into the classification of Eocene GSSCP, particularly distinguishing between woody and herbaceous bamboos. Milon et al. [35] evaluated the impact of carbonization on the taxonomic signal in cotton seeds at the interspecific level. The ‘taxonomic signal’ refers to the ability of morphological traits to reflect evolutionary relationships and to classify taxa accurately. Using linear measurements and 2D GMM, the researchers compared modern dried cotton seeds of four domesticated cotton species and experimentally charred seeds. They found that while charring caused a reduction in size, there was no significant shape deformation. The ‘outline form’ of seeds refers specifically to the external perimeter or silhouette, which can remain intact even if other internal structures or proportions change, while ‘shape’ encompasses broader morphological traits, including internal proportions and curvatures. The study suggests that the outline form is more effective for distinguishing between taxa rather than their overall shape. This suggests that while shape analysis alone was not sufficient to discriminate species, form analysis, which includes size-related traits, proved more effective for taxonomic classification. However, it also underscores the limitations of this method for taxonomical studies of archaeological cotton seeds, emphasizing the need for further research in this area.

Finally, Poulíčková et al. [59] conducted a study on microalgae cells of the *Micrasterias* genus, examining the evolutionary implications of DNA content variation and correlations with morphometric traits, including cell length and complexity analyzed through GMM. Their statistical analysis unveiled notable correlations between DNA content and average cell length, as well as the number of terminal lobes, with cell length displaying the most robust correlation.

### 4.2. Plant Adaptation to Different Habitats

The reports included in this topic highlight the GMM contribution to the study of the complex interactions between plant morphology, environmental conditions, and developmental stability across different plant species and geographical regions. They underscore the importance of considering both genetic and environmental factors in shaping leaf morphology and highlight the adaptive capacity of plants to optimize their performance under varying ecological conditions.

Baranov et al. [60] focused on the small-leaved linden (*Tilia cordata* Mill.) leaf blades in two distinct regions, revealing significant differences in leaf size and asymmetry between the northern and southern populations. The use of GMM provided insights into the variability of leaf shape, with the northern population exhibiting higher variability and directional asymmetry (DA). In contrast, Chen et al. [61] explored the leaf shape variability of the Betulaceae *Carpinus tientaiensis* W.C.Cheng to different light environments, demonstrating the species’ adaptive capacity to optimize photosynthetic efficiency. Their findings underscored the correlation between leaf shape changes and environmental factors such as light intensity, temperature, and humidity. Glennon and Cron [62] explored the relationship between leaf morphology and climatic niches in *Helichrysum* species (Asteraceae), revealing patterns of leaf shape variation associated with cold, dry climates. While leaf shape was not significantly correlated with climatic niche across all species, certain trends emerged, particularly in *H. odoratissimum* (L.) Sweet., where smaller, narrower leaves were prevalent in cooler, drier regions. Hou et al. [63] expanded on these insights by investigating leaf shape variation in *Artemisia* species across broad climatic and soil gradients in China. Their findings underscored the significant role of species identity in shaping leaf morphology, while also demonstrating the responsiveness of leaf shape to regional environmental gradients such as precipitation and soil properties. Lastly, Jovanović et al. [64] focused on leaf size and shape differences in *Quercus cerris* L. along environmental gradients in Serbia, revealing significant variations influenced by soil types, exposures, and elevations. Their study highlighted the plasticity of leaf morphological traits in response to habitat differences, with nutrient-deficient soils resulting in smaller leaf size and higher FA. Two more studies on leaf shape and size variation were assigned to the present topic. Both studies highlight the importance of considering genetic differentiation and environmental factors in shaping leaf morphology of woody plants. Li et al. [65] investigated leaf morphology variation in *Quercus aquifolioides* Rehder and E.H.Wilson and found significant differentiations in leaf shape and size among different lineages, with genetic effects primarily explaining the variation. Yang et al. [66] compared the effectiveness of traditional morphometric methods and GMMs in studying morphological characteristics of *Quercus dentata* Thunb. leaves coming from three different sites, demonstrating the higher classification accuracy of GMMs in analyzing leaf morphological variation. Rahmouni et al. [67] assessed different genotypes of the argan tree, *Argania spinosa* L., across various environments in Morocco, revealing significant variations in leaf shape and size among genotypes and locations, with strong genotype–environment interactions. As different genotypes are taken into consideration, biodiversity is another topic that is coherent within these reports.

The following two studies included in this topic were about the complex dynamics of segment morphology and calcification in *Halimeda* populations (calcareous green seaweeds belonging to the Halimedaceae family), highlighting the importance of segment position and shape in determining calcification levels and overall carbonate budget in Mediterranean ecosystems. Neustupa and Nemcova [68] elucidated the relationship between segment morphology, calcification, and spatial factors in *Halimeda tuna* (J. Ellis and Solander) J. V. Lamouroux, 1816 populations along the Adriatic Sea coast. Their study revealed that segment position on the thallus was the primary determinant of shape features, with lower segments exhibiting lower CaCO_3_ content compared to reniform and oval segments. Then, Nemcova et al. [69] investigated segment shape variation and calcification levels in *H. tuna* along a depth transect in the northern Adriatic Sea. They found that while segment shape, size, and asymmetry were not significantly affected by depth, deeper-growing plants exhibited higher levels of calcification, particularly in apical and subapical segments.

Finally, a study on *H. tuna* by Neustupa and Nemcova [70] investigated the relationship between segment shape, size, and asymmetry of the thallus at two locations in the northern Adriatic Sea. They utilized GMM to analyze equidistant semi-landmarks along segment outlines and found that symmetric variation (the variation in shape that occurs without affecting symmetry) was largely influenced by allometry. Allometry refers to the proportional changes in the size of a whole organism or part of it, and in this case, it accounted for observed shape differences among populations. While smaller segments exhibited greater asymmetry, differences in asymmetry among populations suggested additional influences from local environmental factors, making *Halimeda* an intriguing model for studying morphometric symmetry and asymmetry in benthic coastal habitats.

### 4.3. Plant Reshape in Response to Environmental Changes

The following reports deepened the research field of plant phenotyping using GMM methods as the primary tools for investigation. These studies encompass diverse plant species and organs, exploring various aspects of plant morphology in response to environmental factors, including genetic variations and reproductive strategies. Among them, Neustupa et al. [71] examined temperature-related morphological variations in the unicellular green alga *Micrasterias rotata* Ralfs using GMM, revealing significant shape differences in response to temperature changes, highlighting the potential impact of environmental factors on algal populations. Additionally, Neustupa et al. [72] conducted a comprehensive analysis of desmid species (*M. rotata* and *M. fimbriata* Ralfs) using geometric morphometric techniques, revealing phylogenetic and morphological inconsistencies (i.e., both unexpected differences in the evolutionary relationships between populations and irregular variations in their morphological traits) across different geographic regions. Specifically, they found that populations from different regions exhibited morphological differences that did not always correspond to their genetic relationships, suggesting complex interactions between geography, environment, and evolutionary history. This underscores the utility of GMM in elucidating morphological variation and phylogenetic relationships within plant species.

Moving to flower phenotyping, Neustupa and Woodard [73] explored the size differences and shape variation in zygomorphic corollas of *Glechoma hederacea* L., shedding light on the impact of sexual differentiation on bilateral FA in flower shape. Meanwhile, Neustupa [74] broke new ground by applying GMM to analyze floral symmetry in *Euonymus europaeus* L., offering valuable insights into shape disparities between female and bisexual flowers, marking a significant milestone as the first application of GMM in the study of morphological patterns in a sexually differentiated plant system. Additionally, Tucić et al. [75] investigated the influence of solar irradiance on flower morphology in Iris pumila, showcasing the pivotal role of phenotypic plasticity in shaping floral asymmetry, thereby expanding our understanding of how environmental cues shape plant phenotypes. In a different ecological context, Rosas et al. [76] investigated intraspecific variations in flower shape and color in *Mammillaria haageana* Pfeiff. using GMM, underscoring the potential of geometric morphometric approaches in unraveling the intricate interplay between flower phenotype variation and solar radiation factor. Similarly, Zhao and Schoen [77] studied ecotypes’ variations in *Impatiens capensis* Meerb. to assess the impact of relaxed selection on chasmogamous flowers, comparing sunny and shady populations. Despite expectations, geometric morphometric analysis showed minimal differences in the flower sepal shape among ecotypes, implying potential pleiotropic effects of mutations. However, shaded ecotype populations exhibited significantly smaller sepals, suggesting directional mutational effects towards reduced size, which might be disadvantageous under sunnier conditions where chasmogamous flowers prevail.

Shifting the focus to leaf phenotyping in different environmental situations, Manacorda and Asurmendi [78] analyzed shape variations in *Arabidopsis rosette* images during viral infection processes using GMM, demonstrating the efficacy of geometric morphometric tools in quantifying shape differences between control and infected plants, showcasing the potential of GMM in enhancing the extraction and processing of phenotypic features objectively and reproducibly. Additionally, Stefanovic et al. [79] investigated needle morphology and intraspecific variability in *Taxus baccata* L. populations using GMM, revealing sexual dimorphism and phenotypic plasticity in response to different bioclimatic factors.

The last report assigned to this topic involved GMM analyses on three plant organs: the stem, leaf, and flower of *Teucrium montanum* L. [80]. Authors highlighted differences in the size and shape of morphological traits in mountain germander populations from different geological substrates using GMM, emphasizing the significance of shape–environment relationships in plant adaptation (also related to the topic, plant adaptation to different habitats).

### 4.4. Plant Biodiversity

Concerning biodiversity, Van de Kerke et al. [81] proposed a method for quantifying floral shape using GMM on virtual 3D models reconstructed from 2D photographic data, demonstrating its efficacy in capturing shape variation in 90 species of *Pelargonium*. Their results reveal significant differences in floral shape among *Pelargonium* species and within species, emphasizing the importance of incorporating the third dimension to accurately interpret shape variation in flowers. Similarly, Wang et al. [82] used micro-CT and GMM to analyze floral shape variations in florist’s gloxinia (*Sinningia speciose* Baill.), revealing unique insights into flower opening and dorsoventral symmetry that could not be discerned using traditional 2D methods, thereby opening new avenues for investigating floral shape variations.

Most of the selected reports for this topic focused on leaves; in particular, they explored leaf morphology and asymmetry across diverse plant species. The studies employ a range of techniques within GMM, including principal component analysis (PCA), canonical variate analysis (CVA), digital image analysis, and statistical analyses such as t-tests and linear discriminant analysis. This methodological diversity reflects the adaptability of GMM to different research questions and plant systems. Viscosi and Cardini [83] discussed this in their work, presenting a detailed protocol for leaf morphology analysis using GMM, promoting the widespread use of these methods in botanical taxonomy and other fields of biology. The study highlights the efficiency of Procrustes-based methods (i.e., generalized Procrustes analysis) in measuring differences in leaves and reveals small-scale variation among populations of *Quercus petraea* (Matt.) Liebl. Several studies, such as those by Huang and Liu [84] and Viscosi [85], contribute to identify species-specific leaf shape differences in both genera of Quercus and Sagittaria, respectively, and providing insights into the relationship between leaf variation and venation architecture. For example, Huang and Liu [84] observed distinct leaf shape differences between species like *Quercus robur* L. and Quercus petraea, with the former displaying more rounded, broader leaves and the latter exhibiting narrower, longer leaves. Similarly, in the genus Sagittaria, Viscosi [85] identified species-specific leaf morphologies, such as the arrow-shaped leaves of *Sagittaria latifolia* Willd. compared to the more elongated and linear leaves of *Sagittaria pygmaea* Miq. These species-specific differences in leaf shape highlight the importance of leaf morphology and venation patterns in species identification and adaptation to various ecological conditions. The studies by Vander Mijnsbrugge [86,87] both underscore the importance of morphological variation in understanding ecological processes and adaptation in plant populations. The former study focuses on the leaf phenotypic variability and asymmetry of two Alnus species, emphasizing their ecological implications, particularly in hybridization and adaptation to different environments. The latter analyzes *Ulmus laevis* Pall. trees to identify deviating natural populations with unique leaf characteristics. The study suggests that these deviations may indicate ecotype evolution, likely driven by isolation and diminished population sizes. Both studies demonstrate the utility of GMM in elucidating the relationships between morphology, genetics, and ecology. Additionally, they highlight the potential of these methods in detecting individual traits affected by introgression, divergence, and environmental pressures. Several studies, such as those by Schmidt and Kahlen [88] and Viscosi et al. [89,90,91], demonstrate the practical applications of GMM in fields such as plant modeling and genetic analysis. These studies highlight the potential for GMM to inform practical applications in agriculture, conservation, and ecosystem management. The studies by Baranov, de Morais et al., and Lexer et al. [92,93,94] collectively demonstrate the power of GMM in understanding plant morphology and its ecological and evolutionary implications. Baranov [92] shed light on asymmetry in *Betula pendula* Roth. leaves, revealing significant asymmetry at various levels of the ecosystem. The study used Generalized Procrustes Analysis to examine leaf morphology and asymmetry within populations, concluding that the observed asymmetry could be attributed to both genetic and environmental factors. The findings emphasize the importance of understanding morphological asymmetry in plants, as it can reflect ecological and developmental processes, providing insights into the adaptability and evolutionary patterns of plant populations. Similarly, de Morais et al. [93] utilized leaf GMM to describe leaf shape variation in *Dalbergia ecastaphyllum* (L.) Taub. populations in Brazil. Their analysis identified significant variations in leaf shape across different populations, highlighting differences in leaf size, shape, and venation patterns. These variations were linked to environmental factors such as habitat conditions and local climate, demonstrating how GMM can be effectively used to characterize variation in leaf morphology even at the population level. Lexer et al. [94] focused on a hybrid zone between *Populus alba* L. and *P. tremula* L., demonstrating the effectiveness of GMM in detecting individual traits influenced by introgression and divergence. Specifically, they identified differences in leaf shape, size, and petiole length, which were influenced by both species’ genetic contributions. These traits were key in distinguishing hybrid individuals from parental species and in revealing the morphological consequences of gene flow and divergence between the two species. Together, these studies underscore the versatility and significance of GMM in elucidating patterns of morphological variation in plants. By providing detailed analyses of leaf morphology and its ecological and evolutionary implications, they contribute to our understanding of plant adaptation, hybridization, and evolution in diverse ecological contexts. Vergara et al. [95] examined phytochemistry, reproductive traits, and growth architecture correlations in *Cannabis* hybrids using GMM methods, challenging traditional taxonomy and suggesting the need for a new system to identify variation within *Cannabis*.

Sakamoto et al. [96] investigated seed shape variations in a sorghum (*Sorghum bicolor*) germplasm collection to explore their association with genome-wide single-nucleotide polymorphisms (SNPs) using genomic prediction (GP) and genome-wide association studies (GWASs). They examined various combinations of shape description methods and standardization procedures, finding that PCA of quantitative descriptors from different methods yielded similar results. In addition, they highlighted the importance of considering methodological factors such as shape description methods and standardization procedures when conducting morphometric analyses of seed shape variations. Their findings emphasize the need for careful consideration and standardization of methodologies to ensure robust and reliable results in studies of this nature.

Finally, in Neustupa’s study [97], cells of the unicellular green algae *Micrasterias compereana* Neustupa, St’astný, and Skaloud, renowned for their complexity, were examined using various geometric morphometric methods to explore their integration and asymmetry. The research revealed that opposite semicells exhibited significant cellular asymmetry, while the second important asymmetry pattern involved differentiation between adjacent lobules within the quadrants. Further analysis indicated complete independence between opposite semicells, weak integration of polar lobes with adjacent lateral lobes, and significant integration between the two lateral lobes of the same semicell. These findings shed light on the intricate morphology of *Micrasterias* cells, with implications for understanding cellular development and adaptation in response to environmental factors.

### 4.5. Plant Development

Only two reports among those selected for this review were exclusively assigned to the ‘Plant development’ topic. The first was the work by Renner et al. [98] on the lobule morphology changes in *Radula* (subgenus *Cladoradula*) liverworts, which revealed rapid morphological change driven by heterochrony. They found that lobule growth duration differences explained a significant portion of the variation in lobule shape among species. The study highlighted the role of heterochronic changes in driving morphological diversification in liverwort species within the subgenus *Cladoradula* clade, contrasting with relatively stable morphology in ancestral nodes. The second work, by Schwallier et al. [99], investigated the ontogeny of upper and lower type pitchers in *Nepenthes rafflesiana* Jack tropical pitcher plants from an anatomical and quantitative morphological perspective. They identified distinct developmental phases and microstructural changes in pitcher development, revealing independent developmental programs for upper and lower pitchers. The study provides insights into the evolutionary adaptations of *Nepenthes* pitcher plants to exploit different food sources through the development of distinct pitcher morphologies.

### 4.6. Anthropic Effect on Plant Morphology

Studies included in this topic shed light on how different environmental factors and stressors (e.g., heavy metals, urbanization, and agronomic practices) derived by human activities can shape plant morphology and provide insights into the interaction between plants and their surroundings.

The first three works assigned to this topic focused on different aspects of flower morphology and pollutant influences. Vujić et al. [100] compared the morphology standards (petals) of *Iris pumila* between polluted and unpolluted environments, while Klisarić et al. [101] investigated the biomonitoring potential of flower asymmetry indices in the same species. In the former study, the length, width, centroid size, and shape of *I. pumila* standards were compared between an urban area in the city of Belgrade and the unpolluted Deliblato Sands Nature Reserve. Significant differences were observed in standard length, centroid size, and shape variation between the two areas, with micro-environmental conditions contributing to diversity within clones. Flowers in the polluted environment exhibited shorter and wider petals compared to those in the unpolluted environment, indicating morphological changes in response to environmental stress. In the latter study, authors used naturally growing clones from the arid steppe habitat of the Deliblato Sands Nature Reserve. Several clones (n = 197) were transplanted to a polluted highway site to assess the impact of environmental stress. Despite analyzing both radially and bilaterally symmetrical flower structures and utilizing a heavily polluted environment, only one asymmetry index showed significant differences on the polluted site, suggesting limited efficiency of flower asymmetry as a biomonitoring method for *I. pumila*, but confirming GMM as efficient approach for biomonitoring parameters affected by environmental alteration. Faure et al. [102] paired the impact of urbanization with pollinator visitation rates and explored their impact on *Impatiens capensis* Meerb. flower shape. Using GMM, they quantified the size and shape of flowers from six populations in the Montreal region, Canada, and found significant correlations between floral characteristics and urbanization levels, as well as pollinator visitation rates. Their results suggest that urbanization impacts flower shape through both abiotic factors and changes in pollinator behavior, highlighting the potential effects of urban environments on floral morphology. The last report on flower organs also fits with the ‘Plant–environment co-evolution’ topic. However, it is included in this paragraph because the majority of the study focuses on results from correlation analyses between shape variables and several abiotic stresses. In fact, in Sandner’s study [38], the significance of symmetry in animal traits and flower shape, indicating developmental instability through FA, is emphasized. Through GMM, the symmetry of flowers in inbred and outbred *Mimulus guttatus* Fisch. ex DC. plants subjected to various stress treatments (i.e., CuSO_4_, cut, nutrient deficiency, flooding, and drought) was analyzed, revealing that FA was influenced by inbreeding and competition rather than abiotic stress. Sandner suggests that the effects of inbreeding and competition on FA differ, with FA decreasing with individual biomass, potentially impacting plant demography and plant–pollinator interactions.

The remaining reports selected and assigned to this topic were related to leaf morphological variation and its response to different environmental stressors [103,104,105,106,107,108]. Baranov et al. [103] investigated leaf asymmetry and shape variation in *Plantago major* L. in response to vehicle emissions and climatic conditions. They found higher FA in roadside populations in 2019 and DA in 2020, with climate influencing leaf shape, but not geographical location or leaf gathering place. A different study from de la Paz Pollicelli et al. [104] studied leaf shape variations in alkaliweed *Cressa truxillensis* Kunth in relation to soil metal concentrations belonging to some polluted sites, observing changes in leaf shape associated with metal stress gradients. They suggest using leaf shape as an early biomarker of contamination stress in marsh plants and that GMM is a useful tool. Two studies were conducted on *Limonium brasiliense* (Boiss.) Kuntze [105,106] to validate leaf shape variations as biomarkers for plants in contaminated soils (either under known concentrations of pollutants or along a soil pollution gradient). They found significant changes in leaf shape induced by lead and salt concentrations, as well as related to soil metal gradients, supporting the use of leaf shape as a practical biomarker for stress in polluted environments. A different work focused only on FA in mountain birch (*Betula pubescens* ssp. *Czerepanovii* [Orl.]) leaves exposed to industrial pollution and experimentally induced stresses (i.e., copper and nickel), finding that FA in birch leaves was not a reliable indicator of environmental stress [107]. Moreover, Vujić and colleagues [108] investigated the impact of recreational disturbance on leaf shape and size in *Mercurialis perennis*, observing significant variations associated with disturbance at both the leaf and whole-plant levels, but no significant sex-specific responses to disturbance in leaf traits.

Finally, the studies conducted by Baranov et al. [109] offer valuable insights into the influence of mineral fertilizer (Nitroammophos—NPK—at different doses) on leaf morphology and asymmetry in two different plant species. Baranov and colleagues highlighted significant DA across winter wheat leaf blades of *Triticum aestivum* L., indicating a mixed asymmetry type, while in a different work [110], they extended this investigation to meadow clover (*Trifolium pretense* L.), revealing a correlation between plastic variability, FA, and shape variation. Moreover, they also explored the effects of fertilizer doses on spring wheat leaf blades of *T. aestivum* [60], demonstrating wider blades in fertilized plants and increased asymmetry values with higher doses, suggesting an impact on plant development homeostasis. Together, these studies underscore the intricate relationship between fertilizer application, leaf morphology, and developmental stability, underlying the relevance of GMM methods to provide valuable insights into agricultural practices and plant adaptation strategies.

### 4.7. Genetic Determinants of Plant Morphology

The three studies assigned to this topic collectively demonstrate the utility of GMM in studying floral shape variation and its genetic basis. Berger et al. [111] investigated the impact of gene silencing on floral asymmetry in *Fedia graciliflora* Fisch. and C.A.Mey., finding that knockdowns of specific genes led to morphological changes, particularly in FgCYC2A knockdowns, resulting in more actinomorphic-like flowers. This underscores the role of genes in shaping floral symmetry and suggests unintended effects on floral size and shape. Hsu et al. [112] used GMM to analyze floral shape variations in *Sinningia speciose* Baill., revealing symmetric variation and tube dilation as key contributors to shape changes. They identified strong associations between corolla symmetry and tube dilation and CYCLOIDEA marker alleles, highlighting the utility of GMM in detecting genetic associations in complex floral traits. In a subsequent study, Hsu et al. [113] employed 3D micro-CT and GMM to study petal form variation in *S. speciosa* hybrids, discovering genotype–phenotype associations between petal form and CYCLOIDEA2-like alleles. This study suggests that the transition from zygomorphic to actinomorphic flowers may involve dorsal petal ventralization, emphasizing the importance of 3D GMM in elucidating floral evolution.

Bersweden et al. [114] investigated hybridization patterns in several *Orchis* species hybrid zones using molecular and morphological data, focusing on *O. militaris*–*O. purpurea*, *O. purpurea*–*O. simia*, and *O. anthropophora*–*O. simia* combinations. They found asymmetric backcrossing towards *O. militaris* L., limited hybrid generations in the other combinations, and strong correlations between labellum shape data and nuclear microsatellite data, indicating tension zones with a balance between gene flow and selection.

Two different studies, assigned to this topic, investigated relationships between genetic determinants and leaf characteristics. The first, from Detcharoen et al. [115], explored the antibacterial and anti-inflammatory properties of *Rhodomyrtus tomentosa* (Aiton) Hassk., highlighting its potential in various applications. They conducted genome sequencing and population differentiation analysis using molecular markers and GMM, revealing significant differences in leaf size and shape across locations, even if a correlation between leaf morphology and antibacterial and anti-inflammatory properties requires further studies. The second, from Rejlová et al. [116], investigated the *Urtica dioica* L. complex using morphological and molecular analyses, revealing distinct clusters of diploid subspecies, but no genetic structure, with tetraploids coalescing with diploids. This disparity suggests potential factors such as local adaptation, recent genetic diversification, and hybridization events driving the observed patterns. Finally, Kerstens et al. [117] compared root system architecture responses in *Arabidopsis thaliana* (L.) Heynh. mutants grown on agar-based media versus potting soil, finding genotype-dependent differences in secondary root density and contribution to root system architecture between growth conditions. These studies highlighted the importance of integrating molecular and morphological approaches to understand hybridization, species differentiation, and plant response to environmental conditions.

## 5. Conclusions

Geometric morphometrics offers a transformative approach to studying plant morphology, providing a powerful tool to explore the diversity and complexity of plant forms. In the context of plant–environment co-evolution, significant findings reveal the influence of floral shape on pollinator preferences and differences in petal shapes linked to various pollinator types. Plant adaptation to different habitats is reflected in specific changes in the shapes, sizes, and other morphological traits of plant organs, especially leaves, which are tied to environmental conditions. Moreover, plants undergo morphological reshaping in response to environmental changes, with climate change and other environmental stresses leading to notable variations in shape and size. Geometric morphometrics has also been instrumental in studying plant biodiversity, uncovering the extent of morphological variation both within and between species across ecosystems, thus contributing to a deeper understanding of biodiversity. Regarding plant development, GMM research mainly focuses on ontogenetic changes and developmental processes, particularly how plants develop and adapt morphologically throughout their life cycle. Anthropogenic factors, such as pollution and land use changes, significantly affect plant morphology, especially in terms of shape and size. Additionally, studies have also identified genetic determinants of plant morphology, with various examples demonstrating how genetic factors influence shape and size variations in different plant species. This review highlights the emerging interest in ‘Plant–environment co-evolution,’ which has been extensively studied, while ‘Plant development’ remains an under-represented topic with limited research in this area. In particular, the influence of environmental changes on plant morphology and the genetic basis of these adaptations are both research areas that warrant further exploration. Despite its advantages, GMM also presents certain limitations when applied to plant biology. One major challenge is the correct identification of homologous landmarks, which is often more straightforward in animals, but can be difficult in plants due to the continuous and highly variable nature of plant structures. Many plant organs, such as leaves and petals, lack discrete homologous points, requiring the use of semi-landmarks or outline-based approaches, which may introduce additional variability and require specialized analytical techniques. Furthermore, GMM is highly dependent on sample quality and image acquisition conditions, with factors such as perspective distortions, resolution inconsistencies, and positional errors affecting the accuracy of shape analyses. The method also does not inherently account for functional and developmental aspects of morphology, meaning that additional ecological, genetic, and physiological data are necessary to fully interpret shape variation. Finally, while GMM provides powerful shape quantification, integrating its results with traditional morphometric approaches, genomic data, and ecological modeling remains an area requiring further methodological development. By summarizing the state of knowledge, this review underlines the applicative potentialities of GMM, which is still an under-used methodological approach in plant biology research fields. Starting with this, the study aims to establish a foundation for further research and exploration involving GMM as a useful research tool.

## Data Availability

No new data were proposed with the present work.

## Supplementary Materials

The following supporting information can be downloaded at: https://www.mdpi.com/article/10.3390/plants14050808/s1, Table S1: Complete list of selected reports and related collected data for this review.
