# Supplementary material for: Unveiling Nature’s Architecture: Geometric Morphometrics as an Analytical Tool in Plant Biology
Source: Plants (Basel). 2025 Mar 5;14(5):808. doi: 10.3390/plants14050808 (PMC11901851; doi:10.3390/plants14050808)
Supplement: Supplementary file 1 [file plants-14-00808-s001.zip › plants-3445515-supplementary.pdf]

Supplementary Material Table S1. Complete list of selected reports and related collected data for this review. For each report, different information has been record taxonomical level), investigated plant organs, objectives investigated through Geometric Morphometrics (GMM), 2D or 3D imaging applied, number of fixed la involved in GMM analyses, main topic the report has been assigned to.

N	Article	Species	Investigated plant organs	GMM objectives	2D/3D imaging	N° of L	N° of semi-L
1	Baranov 2018	*Betula pendula*	Leaf	Asymmetry	2D	12	-
2	Baranov et al., 2020a	*Triticum aestivum*	Leaf	Shape variation and asymmetry	2D	2	48
3	Baranov et al., 2020b	*Trifolium pratense*	Leaf	Shape variation and asymmetry	2D	10	50
4	Baranov et al., 2021a	*Tilia cordata*	Leaf	Shape variation and asymmetry	2D	50	-
5	Baranov et al., 2021b	*Plantago major*	Leaf	Shape variation and asymmetry	2D	52	-
6	Baranov et al., 2021c	*Triticum aestivum*	Leaf	Shape variation and asymmetry	2D	2	50
7	Barišić Klisarić et al., 2019	*Iris pumila*	Flower	Perianth asymmetry: standard_fall_style	2D	2_2_2	11_11_7
8	Benitez-Vieyra et al., 2009	*Bipinnula penicillata*	Flower	Labellum shape	2D	5	4
9	Berger et al., 2017	*Fedia graciliflora*	Flower	Corolla shape variation	2D	10	-
10	Bersweden et al., 2021	*O. militaris–O. purpurea; O. purpurea–O. simia; O. anthropophora–O. simia*	Flower	Labellum shape	2D	15	-
11	Bilbao et al., 2021	*Erythrina* genus	Flower	Petal shape	2D	3	40
12	Budečević et al., 2023	*Iris pumila*	Flower	outer_inner perianth shape_style branch shape variation and asymmetry	2D	18_19 _18	-
13	Chen et al., 2018	*Carpinus tientaiensis*	Leaf	Shape variation	2D	17	-
14	de la Paz Pollicelli et al., 2018	*Cressa truxillensis*	Leaf	Shape and size variation	2D	3	10
15	de Morais et al., 2019	*Dalbergia ecastaphyllum*	Leaf	Shape variation	2D	2	14
16	Detcharoen et al., 2023	*Rhodomyrtus tomentosa*	Leaf	Shape variation	2D	4	-
17	Faure et al., 2022	Gesneriaceae (18 species)	Flower	Corolla shape variation and curvature	2D	6	13

18	Faure et al., 2023	*Impatiens capensis*	Flower	Front_profile shape variation	2D	8_6	52_40
19	Gallaher et al., 2019	Poaceae	Leaf	Shape and size variation	2D	2	100
20	Gallaher et al., 2020	Poaceae subfamilies	GSSC phytoliths	Shape and size variation	3D	6	-
21	García et al., 2020	*Nicotiana glauca*	Flower	Corolla shape variation and length	2D	14	4
22	Glennon and Cron, 2015	*Helichrysum odoratissimum* ; *H. griseolanatum* ; *H.*	Leaf	Shape variation	2D	12	10
23	Gómez et al., 2014	*Erysimum* genus	Flower	Corolla shape variation	2D	32	-
24	Gómez et al., 2016	Brassicaceae	Flower	Corolla shape variation	2D	32	-
25	Hanušová et al., 2014	*Diphasiastrum*	Stem	Dorsal_ventral side shape variation	2D	9_9	28_28
26	Hou et al., 2023	*Artemisia* genus	Leaf	Shape variation	2D	2	38
27	Hsu et al., 2015	*Sinningia speciosa*	Flower	Front_profile shape variation	2D	5_5	25_10
28	Hsu et al., 2017	*Sinningia speciosa*	Flower	Petal shape, size and L-R asymmetry	3D	7	25
29	Hsu et al., 2020	*Sinningia* genus	Flower	Corolla shape and size variation	3D	25	390
30	Huang and Liu, 2014	*Sagittaria* genus	Leaf	Shape variation	2D	6	6
31	Ibañez et al., 2023	*Jaborosa* genus	Flower	Frontal_sagittal shape and size variation	2D	3_11	6_4
32	Idaszkin et al., 2019	*Limonium brasiliense*	Leaf	Shape and size variation	2D	5	20
33	Idaszkin et al., 2023	*Limonium brasiliense*	Leaf	Shape and size variation	2D	5	20
34	Joly et al., 2018	Gesneriinae subtribe	Flower	Corolla shape variation	2D	6	26
35	Jovanović et al., 2023	*Quercus cerris*	Leaf	Shape and size variation	2D	13	-
36	Kerstens et al., 2021	*Arabidopsis thaliana*	Root	Shape variation	2D	5	-
37	Lexer et al., 2009	*Populus alba* , *P. tremula*	Leaf	Shape variation	2D	2	-
38	Li et al., 2021	*Quercus aquifolioides*	Leaf	Shape and size variation	2D	13	-

39	Manacorda and Asurmendi, 2018	*Arabidopsis thaliana*	Leaf	Shape and size rosette variation	2D	11	-
40	McCarthy et al., 2016	*Nicotiana* genus	Flower	Shape variation	2D	15	-
41	McCarthy et al., 2019	*Nicotiana* genus	Flower	Shape variation	2D	15	-
42	Milon et al., 2023	*Gossypium arboreum* , *G. herbaceum* , *G. barbadense* , *G. hirsutum*	Seed	Shape and size variation	2D	1	49
43	Nemcova et al., 2023	*Halimeda tuna*	Thallus	Shape and size segment variation	2D	1	79
44	Neustupa and Nemcova, 2018	*Halimeda tuna*	Thallus	Shape/size segment variation and asymmetry	2D	1	89
45	Neustupa and Nemcova, 2022	*Halimeda tuna*	Thallus	Shape and size segment variation	2D	1	79
46	Neustupa and Woodard, 2021	*Glechoma hederacea*	Flower	Corolla shape/size variation and asymmetry	2D	5	116
47	Neustupa et al., 2008	*Micrasterias rotata*	Cell	Shape and size semi-cell variation	2D	31	-
48	Neustupa et al., 2011	*Micrasterias fimbriata* , *M. rotata*	Cell	Shape and size semi-cell variation	2D	49	-
49	Neustupa, 2017	*Micrasterias compereana*	Cell	Quadrants/terminal lobule symmetry	2D	208	-
50	Neustupa, 2020	*Euonymus europaeus*	Flower	Corolla shape and size variation	2D	8	192
51	O'hanlon et al., 2014	Different species	Flower	Petal shape and size variation	2D	2	22
52	Poulíèková et al., 2014	*Micrasterias* genus	Cell	Shape and size variation	2D	40	-
53	Rahmouni et al., 2020	*Argania spinosa*	Leaf	Shape and size variation	2D	61	-
54	Rejlová et al., 2020	*Urtica dioica*	Leaf	Shape variation	2D	6	50
55	Renner et al., 2013	*Cladoradula* subgenus	Leaf	Shape lobule variation	2D	4	34
56	Rosas et al., 2022	*Mammillaria haageana*	Flower	Tepal shape variation	2D	3	7
57	Rubini Pisano et al., 2019	*Mandevilla laxa* ; *M. pentlandiana*	Flower	Shape variation	2D	13	-
58	Sakamoto et al., 2019	*Sorghum bicolor*	Seed	Shape and size variation	2D	1	499
59	Sandner et al., 2019	*Betula pubescens* ssp. *Czerepanovii*	Leaf	Asymmetry	2D	5	-

60	Sandner, 2020	*Mimulus guttatus*	Flower	Asymmetry	2D	6	-
61	Schmidt and Kahlen, 2018	*Cucumis sativus*	Leaf	Shape variation and asymmetry	3D	17	-
62	Schnablová et al., 2020	110 Herbaceus plant species	Shoot apical meristem (SAM)	Shape variation	2D	2	98
63	Schwallier et al., 2020	*Nepenthes rafflesiana*	Leaf	Shape variation	3D	18	-
64	Stefanovic et al., 2017	*Taxus baccata*	Leaf	Shape and size variation	2D	3	22
65	Strelin et al., 2016	14 Loasoideae species	Flower	Corolla/staminodial complex shape and size	2D	ND	ND
66	Strelin et al., 2021	*Calceolaria polyrhiza*	Flower	Corolla shape and size variation	2D	8	-
67	Tucić et al., 2018	*Iris pumila*	Flower	Shape, size variation and asymmetry (fall/standard/style	2D	18_19 _18	-
68	van de Kerke et al., 2020	*Pelargonium* Genus	Flower	Tube/petal shape variation	2D	10_30	75
69	Vander Mijnsbrugge 2015	*Alnus glutinosa* , *A. incana*	Leaf	Shape and size variation	2D	11	-
70	Vander Mijnsbrugge 2016	*Ulmus laevis*	Leaf	Shape and size variation	2D	13	-
71	Vergara et al., 2021	*Cannabis sativa*	Leaf	Shape variation	2D	10	-
72	Viscosi 2015	*Quercus frainetto* , *Q. petraea* and *Q. pubescens*	Leaf	Shape, size variation and asymmetry	2D	13	-
73	Viscosi and Cardini, 2011	*Quercus petraea*	Leaf	Right leaf side shape and size variation	2D	11	-
74	Viscosi et al., 2009a	*Quercus robur* , *Q. petraea* , *Q. frainetto* and *Q. pubescens*	Leaf	Right leaf side shape variation	2D	11	-
75	Viscosi et al., 2009b	*Quercus petraea* , *Q. robur* , *Q. pubescens* , *Q. pyrenaica*	Leaf	Right leaf side shape variation	2D	11	-
76	Viscosi et al., 2012	*Quercus frainetto* , *Q. petraea* and *Q. pubescens*	Leaf	Right leaf side shape and size variation	2D	11	-
77	Vujić et al., 2015	*Iris pumila*	Flower	Corolla shape and size variation	2D	2	14
78	Vujić et al., 2016	*Mercurialis perennis*	Leaf	Shape variation	2D	2	10
79	Wang et al., 2015	*Sinningia speciosa*	Flower	Shape variation	3D	20	75

80	Wolcott et al., 2023	*Theobroma cacao*	Flower	Shape and size variation	3D	-	135
81	Yang et al., 2022	*Quercus dentata*	Leaf	Shape variation	2D	13	-
82	Zhao and Schoen, 2022	*Impatiens capensis*	Flower	Sepal shape and size variation	2D	8	24
83	Zlatic et al., 2023	*Teucrium montanum*	Stem, Leaf, Flower	Stem_Leaf_Corolla shape and size variation	2D	12_8_ 18	-

References

Baranov, S. G. (2018). Morphometric analyses of (hidden) directional asymmetry in leaf blades. Emerging Science Journal, 2(4), 170-180.

Baranov, G., Vinokurov, I. Y., Zykov, I. E., Fedorova, L. V., & Antsyshkina, A. M. (2021c). Two kinds of asymmetry in spring wheat leaf blade. In IOP Confe

Baranov, S. G., Vinokurov, I. Y., Schukin, I. M., Schukina, V. I., Malcev, I. V., Zykov, I. E., Ananieff, A. A., Fedorova, L. V. (2020a). Does Fertilizer Influenc

Baranov, S. G., Vinokurov, I. Y., Zykov, I. E., & Fedorova, L. V. (2020b). Asymmetry and shape in leaf blade red clover. In IOP Conference Series: Earth and

Baranov, S. G., Zykov, I. E., Biryukova, T. S., Fedorova, L. V., & Antsyshkina, A. M. (2021b). About the asymmetry structure of the leaf blade Common plant

Baranov, S. G., Zykov, I. E., Poloskova, E. Y., Lipponen, I. N., Goncharova, O. A., & Kuznetsova, D. D. (2021a). Spatial Variability of Small-leaved Linden (T

Barišić Klisarić, N., Miljković, D., Avramov, S., Živković, U., & Tarasjev, A. (2019). Radial and bilateral fluctuating asymmetry of Iris pumila flowers as indic

Benitez-Vieyra, S., Medina, A. M., & Cocucci, A. A. (2009). Variable selection patterns on the labellum shape of Geoblasta pennicillata, a sexually deceptive o

Berger, B. A., Ricigliano, V. A., Savriama, Y., Lim, A., Thompson, V., & Howarth, D. G. (2017). Geometric morphometrics reveals shifts in flower shape sym

Bersweden, L., Viruel, J., Schatz, B., Harland, J., Gargiulo, R., Cowan, R. S., ... & Fay, M. F. (2021). Microsatellites and petal morphology reveal new patterns

Bilbao, G., Bruneau, A., & Joly, S. (2021). Judge it by its shape: a pollinator-blind approach reveals convergence in petal shape and infers pollination modes in

Budečević, S., Hočevar, K., Manitašević Jovanović, S., & Vuleta, A. (2023). Phenotypic Selection on Flower Traits in Food-Deceptive Plant Iris pumila L.: The

Chen, M., Jin, Z., & Ke, S. (2018). Measurement and analysis of leaf shape variation of Carpinus tientaiensis in different light environment. Scientia Silvae Sin

de la Paz Pollicelli, M., Idaszkin, Y. L., Gonzalez-José, R., & Márquez, F. (2018). Leaf shape variation as a potential biomarker of soil pollution. Ecotoxicology

Detcharoen, M., Bumrungsri, S., & Voravuthikunchai, S. P. (2023). Complete Genome of Rose Myrtle, Rhodomyrtus tomentosa, and Its Population Genetics in

Faure, J., Martén-Rodríguez, S., Clark, J. L., & Joly, S. (2022). The level of pollination specialization affects the relationship between the shape of flowers and

Faure, J., Volz, V., & Joly, S. (2023). Variation in flower size and shape of Impatiens capensis is correlated with urbanization in Montreal, Canada. Ecology and

Gallaher, T. J., Adams, D. C., Attigala, L., Burke, S. V., Craine, J. M., Duvall, M. R., ... & Clark, L. G. (2019). Leaf shape and size track habitat transitions acro

Gallaher, T. J., Akbar, S. Z., Klahs, P. C., Marvet, C. R., Senske, A. M., Clark, L. G., & Strömberg, C. A. (2020). 3D shape analysis of grass silica short cell ph

García, M., Benítez-Vieyra, S., Sérsic, A. N., Pauw, A., Cocucci, A. A., Traveset, A., ... & Paiaro, V. (2020). Is variation in flower shape and length among nati

Glennon, K. L., & Cron, G. V. (2015). Climate and leaf shape relationships in four Helichrysum species from the Eastern Mountain Region of South Africa. Ev

Gómez, J. M., Perfectti, F., & Klingenberg, C. P. (2014). The role of pollinator diversity in the evolution of corolla-shape integration in a pollination-generalist

Gómez, J. M., Torices, R., Lorite, J., Klingenberg, C. P., & Perfectti, F. (2016). The role of pollinators in the evolution of corolla shape variation, disparity and

Hanušová, K., Ekrt, L., Vit, P., Kolář, F., & Urfus, T. (2014). Continuous morphological variation correlated with genome size indicates frequent introgressive

Hou, X., Gao, R., Huo, L., Yu, X., & Yang, X. (2023). Geographical variation of Artemisia leaf morphology along a large environmental gradient in China. Pla
Hsu, H. C., Chen, C. Y., Lee, T. K., Weng, L. K., Yeh, D. M., Lin, T. T., ... & Kuo, Y. F. (2015). Quantitative analysis of floral symmetry and tube dilation in a
Hsu, H. C., Chou, W. C., & Kuo, Y. F. (2020). 3D revelation of phenotypic variation, evolutionary allometry, and ancestral states of corolla shape: a case study
Hsu, H. C., Wang, C. N., Liang, C. H., Wang, C. C., & Kuo, Y. F. (2017). Association between petal form variation and CYC2-like genotype in a hybrid line of
Huang, L. J., & Liu, Y. C. (2014). Understanding diversity in leaf shape of Chinese sagittaria (Alismataceae) by geometric tools. Pak J Bot, 46(6), 1927-1934.
Ibañez, A. C., Sérsic, A. N., Cocucci, A. A., & Moré, M. (2023). Flower morphology evolution in Jaborosa (Solanaceae): shape and size variation associated wi
Idaszkin, Y. L., de la Paz Pollicelli, M., & Márquez, F. (2023). Assessment of halophyte plant phenotypic responses under heavy metals pollution. Implications
Idaszkin, Y. L., Márquez, F., Mateos-Naranjo, E., de la Paz Pollicelli, M., & Cisneros, H. S. (2019). Multidimensional approach to evaluate Limonium brasilien
Joly, S., Lambert, F., Alexandre, H., Clavel, J., Léveillé-Bourret, É., & Clark, J. L. (2018). Greater pollination generalization is not associated with reduced con
Jovanović, M., Milovanović, J., Nonić, M., Šijačić-Nikolić, M., Kerkez Janković, I., & Grbović, F. (2023). The Effects of Soil Type, Exposure and Elevation on
Kerstens, M., Hesen, V., Yalamanchili, K., Bimbo, A., Grigg, S., Opdenacker, D., ... & Willemsen, V. (2021). Nature and nurture: genotype-dependent differen
Lexer, C., Joseph, J., van Loo, M., Prenner, G., Heinze, B., Chase, M. W., & Kirkup, D. (2009). The use of digital image -based morphometrics to study the phe
Li, Y., Zhang, Y., Liao, P. C., Wang, T., Wang, X., Ueno, S., & Du, F. K. (2021). Genetic, geographic, and climatic factors jointly shape leaf morphology of an
Manacorda, C. A., & Asurmendi, S. (2018). Arabidopsis phenotyping through geometric morphometrics. GigaScience, 7(7), giy073.
McCarthy, E. W., Chase, M. W., Knapp, S., Litt, A., Leitch, A. R., & Le Comber, S. C. (2016). Transgressive phenotypes and generalist pollination in the floral
McCarthy, E. W., Landis, J. B., Kurti, A., Lawhorn, A. J., Chase, M. W., Knapp, S., ... & Litt, A. (2019). Early consequences of allopolyploidy alter floral evolu
Milon, J., Bouchaud, C., Viot, C., Lemoine, M., & Cucchi, T. (2023). Exploring the carbonization effect on the interspecific identification of cotton (Gossypium
Morais, D. V. D., Nunes, L. A., Mata, V. P. D., Costa, M. A. P. D. C., Sodre, G. D. S., & Carvalho, C. A. L. D. (2019). Leaf geometric morphometrics among p
Nemcova, Y., Orlando-Bonaca, M., & Neustupa, J. (2023). Halimeda tuna (Bryopsidales, Ulvophyceae) calcification on the depth transect in the northern Adria
Neustupa, J. (2017). Asymmetry and integration of cellular morphology in Micrasterias compereana. BMC Evolutionary Biology, 17, 1-16.
Neustupa, J. (2020). Gynodioecy in the common spindle tree (Euonymus europaeus L.) involves differences in the asymmetry of corolla shapes between sexual
Neustupa, J., & Nemcova, Y. (2018). Morphological allometry constrains symmetric shape variation, but not asymmetry, of Halimeda tuna (Bryopsidales, Ulvo
Neustupa, J., & Nemcova, Y. (2022). Geometric morphometrics shows a close relationship between the shape features, position on thalli, and CaCO3 content o
Neustupa, J., & Woodard, K. (2021). Male sterility significantly elevates shape variation and fluctuating asymmetry of zygomorphic corolla in gynodioecious G

Neustupa, J., Šťastný, J., Nemjová, K., Mazalová, P., Goodyer, E., Poulíčková, A., & Škaloud, P. (2011). A novel, combined approach to assessing species deli
O'hanlon, J. C., Holwell, G. I., & Herberstein, M. E. (2014). Predatory pollinator deception: Does the orchid mantis resemble a model species?. Current Zoolog
Pouliekova, A., Mazalová, P., Vašut, R. J., Šarhanová, P., Neustupa, J., & Škaloud, P. (2014). DNA content variation and its significance in the evolution of the
Rahmouni, I., Oumouss, S., Tobi, G., Bendaou, N., Bouksaim, M., & El Bahloul, Y. (2020). Phenotypic plasticity of leaf shape in selected and semi-domesticat
Rejlova, L., Böhmová, A., Chumova, Z., Hořčicová, Š., Josefiová, J., Schmidt, P. A., ... & Chrtek, J. (2021). Disparity between morphology and genetics in Urt
Renner, M. A., Devos, N., Brown, E. A., & von Konrat, M. J. (2013). Three modes of heterochrony explain lobule diversity in Radula subgenus Cladoradula (P
Rosas, U., Fuentes-Pérez, E. S., Cervantes, C. R., Sandoval-Zapotitla, E., Santiago-Sandoval, I., Arias, S., & Reyes-Santiago, J. (2022). Evolution of flower allo
Rubini Pisano, A., More, M., Cisternas, M. A., Raguso, R. A., & Benitez-Vieyra, S. (2019). Breakdown of species boundaries in Mandevilla: floral morphologi
Sakamoto, L., Kajiya-Kanegae, H., Noshita, K., Takanashi, H., Kobayashi, M., Kudo, T., ... & Iwata, H. (2019). Comparison of shape quantification methods fo
Sandner, T. M. (2020). Inbreeding and competition, but not abiotic stresses, increase fluctuating asymmetry of Mimulus guttatus flowers. Biological Journal of
Sandner, T. M., Zverev, V., & Kozlov, M. V. (2019). Can the use of landmarks improve the suitability of fluctuating asymmetry in plant leaves as an indicator o
Schmidt, D., & Kahlen, K. (2018). Towards more realistic leaf shapes in functional-structural plant models. Symmetry, 10(7), 278.
Schnablová, R., Neustupa, J., Woodard, K., Klimešová, J., & Herben, T. (2020). Disentangling phylogenetic and functional components of shape variation amo

Schwallier, R., van Wely, V., Baak, M., Vos, R., van Heuven, B. J., Smets, E., ... & Gravendeel, B. (2020). Ontogeny and anatomy of the dimorphic pitchers of
Stefanović, M., Nikolić, B., Matić, R., Popović, Z., Vidaković, V., & Bojović, S. (2017). Exploration of sexual dimorphism of Taxus baccata L. needles in natu
Strelin, M. M., Benitez-Vieyra, S., Fornoni, J., Klingenberg, C. P., & Cocucci, A. A. (2016). Exploring the ontogenetic scaling hypothesis during the diversifica
Strelin, M. M., Cosacov, A., Chalcoff, V. R., Maubecin, C. C., Sérsic, A. N., & Benitez-Vieyra, S. M. (2021). The role of ontogenetic allometry and nonallome
Tucić, B., Budečević, S., Manitašević Jovanović, S., Vuleta, A., & Klingenberg, C. P. (2018). Phenotypic plasticity in response to environmental heterogeneity
van de Kerke, S. J., van Engelenhoven, T., van Es, A. L., Schat, L., van Son, L. M., Vink, S., ... & Bakker, F. T. (2020). Capturing variation in floral shape: a vi
Vander Mijnsbrugge, K. (2015). Morphological dissection of leaf, bud and infructescence traits of the interfertile native A. glutinosa and non-native A. incana i
Vander Mijnsbrugge, K., Le Clercq, R., & Michiels, B. (2016). Dissection of leaf morphological traits from isolated and declined relict populations of Ulmus la
Vergara, D., Feathers, C., Huscher, E. L., Holmes, B., Haas, J. A., & Kane, N. C. (2021). Widely assumed phenotypic associations in Cannabis sativa lack a sha
Viscosi, V. (2015). Geometric morphometrics and leaf phenotypic plasticity: assessing fluctuating asymmetry and allometry in European white oaks (Quercus).
Viscosi, V., & Cardini, A. (2011). Leaf morphology, taxonomy and geometric morphometrics: a simplified protocol for beginners. PloS one, 6(10), e25630.
Viscosi, V., Antonecchia, G., Lepais, O., Fortini, P., Gerber, S., & Loy, A. (2012). Leaf shape and size differentiation in white oaks: assessment of allometric re
Viscosi, V., Fortini, P., Slice, D. E., Loy, A., & Blasi, C. (2009a). Geometric morphometric analyses of leaf variation in four oak species of the subgenus Querc
Viscosi, V., Lepais, O., Gerber, S., & Fortini, P. (2009b). Leaf morphological analyses in four European oak species (Quercus) and their hybrids: A comparison
Vujić, V., Avramov, S., Tarasjev, A., Barišić Klisarić, N., Živković, U., & Miljković, D. (2015). The effects of traffic-related air pollution on the flower morpho
Vujić, V., Rubinjoni, L., Selaković, S., & Cvetković, D. (2016). Small-scale variations in leaf shape under anthropogenic disturbance in dioecious forest forb M
Wang, C. N., Hsu, H. C., Wang, C. C., Lee, T. K., & Kuo, Y. F. (2015). Quantifying floral shape variation in 3D using microcomputed tomography: a case stud
Wolcott, K. A., Stanley, E. L., Gutierrez, O. A., Wuchty, S., & Whitlock, B. A. (2023). 3D pollination biology using micro-computed tomography and geometr
Yang, K., Wu, J., Li, X., Pang, X., Yuan, Y., Qi, G., & Yang, M. (2022). Intraspecific leaf morphological variation in Quercus dentata Thunb.: a comparison of
Zhao, Y., & Schoen, D. J. (2022). Relaxed selection and the evolution of the chasmogamous flower of Impatiens capensis (Balsaminaceae). Evolutionary Ecolo
Zlatić, N., Budečević, S., & Stanković, M. (2023). Geological Substrate Effects on Teucrium montanum L.(Lamiaceae) Morphological Traits: Geometric Morp

ed: progressive number (N), same reference reported in the main text, investigated plant species (or upper
ındmarks (N° of L) and semi-landmarks (N° of semi-L), positioning method used for fixing landmarks, correlates

Positioning of landmarks	Correlates	Topic	Country
Manual	Levels of ecosystem (population, tree, leaf)	Plant biodiversity	Russia
Semi-automatic	Nitroammophos (NPK) different doses	Anthropic effect on plant morphology	Russia
Semi-automatic	Nitroammophos (NPK) different doses	Anthropic effect on plant morphology	Russia
Manual	Latitude	Plant adaptation to different habitats	Russia
Manual	Vehicle emissions	Anthropic effect on plant morphology	Russia
Manual	Nitroammophos (NPK) different doses	Anthropic effect on plant morphology	Russia
Manual	Polluted sites	Anthropic effect on plant morphology	Serbia
Manual	*Capsomeris bistrimacula* body shape	Plant-environment co-evolution	South America
Manual	Virus-induced gene silencing (VIGS)	Genetic determinants of plant morphology	NA
Manual	Nuclear microsatellite markers	Genetic determinants of plant morphology	France
Manual	Pollination modes	Plant-environment co-evolution	NA
Manual	Pollinated/non-pollinated	Plant-environment co-evolution	Serbia
Manual	Irradiance intesity	Plant adaptation to different habitats	China
Manual	Polluted sites	Anthropic effect on plant morphology	South America
Manual	Geographic distances and altitudes	Plant biodiversity	Brazil
Manual	ISSR, SSR markers	Genetic determinants of plant morphology	Thailand
Manual	Hummingbird bill shape and curvature	Plant-environment co-evolution	Greater Antilles

Manual	Urbanization and visitation rates of the pollinators	Anthropic effect on plant morphology	Canada
Manual	Precipitation, solar irradiance, temperature, photosynthetic pathway, habitat	Plant-environment co-evolution	NA
Manual	Phylogenetic tree	Plant-environment co-evolution	NA
Manual	Pollination environments	Plant-environment co-evolution	South America
Manual	Climatic niches	Plant adaptation to different habitats	South Africa
Manual	Pollinators diversity and phylogenetic tree	Plant-environment co-evolution	Europe, Africa
Manual	Phylogenetic tree and pollination niche	Plant-environment co-evolution	NA
Manual	Genome size	Plant-environment co-evolution	Europe
Manual	Climatic and soil gradients	Plant adaptation to different habitats	China
Semi-automatic	SsCYC marker	Genetic determinants of plant morphology	China
Semi-automatic	SsCYC marker	Genetic determinants of plant morphology	China
Semi-automatic	Pollination types and phylogey	Plant-environment co-evolution	China
Manual	Intra- and interspecies	Plant biodiversity	China
Semi-automatic	Pollination modes and phylogeny	Plant-environment co-evolution	South America
Manual	Pb, Pb+NaCl	Anthropic effect on plant morphology	Argentina
Manual	Soil pollution gradient, Pb	Anthropic effect on plant morphology	Argentina
Manual	Pollination strategies and phylogeny	Plant-environment co-evolution	Antilles
Manual	Soil types, expositions and elevations	Plant adaptation to different habitats	Serbia
Manual	CRISPR mutants	Genetic determinants of plant morphology	NA
Manual	Species	Plant biodiversity	Austria
Manual	Geographic, genetic, and climatic factors	Plant adaptation to different habitats	Italy

Manual	Oilseed rape mosaic virus (ORMV); turnip mosaic virus-UK1 strain (TuMV-UK1)	Plant reshape in response to environmental changes	NA
Manual	Allopolyploidy	Plant-environment co-evolution	NA
Manual	Phylogeny	Plant-environment co-evolution	NA
Semi-automatic	Carbonization	Plant-environment co-evolution	France
Semi-automatic	Depth, CaCO3 content	Plant adaptation to different habitats	Slovenia
Semi-automatic	Inter-individual	Plant adaptation to different habitats	Slovenia
Semi-automatic	Spatial factors, segment position, CaCO3 content	Plant adaptation to different habitats	Eastern coast of Adriatic Sea
Semi-automatic	Sex	Plant reshape in response to environmental changes	Czech Republic
Manual	Temperature	Plant reshape in response to environmental changes	Czech Republic
Manual	Plastid-encoded marker (trnG^{ucc})	Plant reshape in response to environmental changes	Czech Republic
Manual	Inter-individual	Plant biodiversity	NA
Semi-automatic	Sex	Plant reshape in response to environmental changes	Czech Republic
Manual	Femoral lobe shape of orchid mantis (*Hymenopus coronatus*)	Plant-environment co-evolution	NA
Semi-automatic	Genome size	Plant-environment co-evolution	Czech Republic
Manual	Genotypes, climatic conditions	Plant adaptation to different habitats	Morocco
Manual	Genotype	Genetic determinants of plant morphology	Europe, Asia
Manual	Time	Plant development	NA
Manual	Solar radiation	Plant reshape in response to environmental changes	NA
Manual	Chemical fragrance pattern	Plant-environment co-evolution	Argentina
Semi-automatic	Inter-individual	Plant biodiversity	NA
Manual	Traditional morphometry, pollution, experimental stress (copper and nickel)	Anthropic effect on plant morphology	Russia

Manual	Pollination, stress ($CuSO_4$, cut, nutrient deficiency, flooding, drought), competition	Anthropic effect on plant morphology; Plant-environment co-evolution	NA
Manual	Prototype leaf shape (*L-Cucumber*)	Plant biodiversity	NA
Semi-automatic	Phylogeny, cell-level variables (genome size, mean cell diameter), plant-level traits	Plant-environment co-evolution	Europe
Manual	4 developmental stages	Plant development	Asia
Manual	Sex, bioclimatic factors	Plant reshape in response to environmental changes	Serbia
Manual	Time, species, pollinators	Plant-environment co-evolution; Development	Germany
Manual	Distance from pollinators, pollinators	Plant-environment co-evolution	South America
Manual	Solar irradiance	Plant reshape in response to environmental changes	Serbia
Manual	Species, virtual3D flower	Plant biodiversity	NA
Manual	Species, inter-individual, morphological	Plant biodiversity	Belgium
Manual	Population, genotype, ramet	Plant biodiversity	Belgium
Manual	Phytochemistry, reproductive traits, growth architecture	Plant biodiversity	NA
Manual	Species	Plant biodiversity	Italy
Manual	Populations, trees, leaves and replicas	Plant biodiversity	Italy
Manual	Environmental factors	Plant biodiversity	Italy
Manual	Genotypes	Plant biodiversity	Italy
Manual	Species	Plant biodiversity	Italy
Manual	Polluted and unpolluted sites	Anthropic effect on plant morphology	Serbia
Manual	Sex, Human trampling	Anthropic effect on plant morphology	Serbia
Manual	Inter-individual	Plant biodiversity	NA

	Manual	Dimensions of pollinators	Plant-environment co-evolution	USA
	Manual	Three provenances	Plant adaptation to different habitats	China
	Manual	Shade and sun ecotypes	Plant reshape in response to environmental changes	Canada
	Manual	Substrates	Plant reshape in response to environmental changes; Plant adaptation to different habitats	Serbia

rence Series: Earth and Environmental Science (Vol. 839, No. 3, p. 032037). IOP Publishing.
e Shape and Asymmetry in Wheat Leaf?. In Artificial Intelligence and Bioinspired Computational Methods:
Environmental Science (Vol. 548, No. 7, p. 072015). IOP Publishing.
ain. In E3S Web of Conferences (Vol. 262, p. 04004). EDP Sciences.ù
Tilia cordata Mill.) Leaf Blade. In E3S Web of Conferences (Vol. 254, p. 06002). EDP Sciences.
cators of environmental stress. Symmetry, 11(6), 818.
rchid. Journal of Evolutionary Biology, 22(11), 2354-2362.
metry and size following gene knockdown of CYCLOIDEA and ANTHOCYANIDIN SYNTHASE. BMC plant
of admixture in Orchis hybrid zones. American journal of botany, 108(8), 1388-1404.
the genus Erythrina. American journal of botany, 108(9), 1716-1730.
e Role of Pollinators. Symmetry, 15(6), 1149.
icae, 54(1), 54-63.
y and Environmental Safety, 164, 69-74.
ı Thai Peninsula. Plants, 12(8), 1582.
the bills of their hummingbird pollinators in Antillean Gesneriaceae. International Journal of Plant Sciences,
d Evolution, 13(12), e10826.
oss forest–grassland boundaries in the grass family (Poaceae). Evolution, 73(5), 927-946.
ytoliths: a new method for fossil classification and analysis of shape evolution. New Phytologist, 228(1), 376-
ive and non-native populations of Nicotiana glauca a product of pollinator-mediated selection?. Evolutionary
olutionary ecology, 29, 657-678.
plant clade. Philosophical Transactions of the Royal Society B: Biological Sciences, 369(1649), 20130257.
integration in a highly diversified plant family with a conserved floral bauplan. Annals of Botany, 117(5), 889-
hybridization among Diphasiastrum species (Lycopodiaceae) in Central Europe. Plos one, 9(6), e99552.

nt Ecology, 1-9.
ın F2 cross of Sinningia speciosa. Scientia Horticulturae, 188, 71-77.
of clade Corytholoma (subtribe Ligeriinae, family Gesneriaceae). GigaScience, 9(1), giz155.
f Sinningia speciosa. Frontiers in Plant Science, 8, 558.

ith contrasting pollination modes. Plant Biology.
for monitoring and phytoremediation. Environmental Pollution, 121916.
ıse as source of early biomarkers for lead pollution monitoring under different saline conditions. Ecological
straints on corolla shape in Antillean plants. Evolution, 72(2), 244-260.
ı Leaf Size and Shape in Quercus cerris L. South-east Eur for, 14(1), 23-08.
tial responses of root architecture to agar and soil environments. Genes, 12(7), 1028.
enotypic mosaic in taxa with porous genomes. Taxon, 58(2), 349-364.
alpine oak, Quercus aquifolioides Rehder & EH Wilson. Annals of Forest Science, 78(3), 1-18.

l evolution of Nicotiana polyploids. Nature Plants, 2(9), 1-9.
ution in Nicotiana (Solanaceae). BMC plant biology, 19(1), 1-19.
ı spp.) seeds using classical and 2D geometric morphometrics. Journal of Archaeological Science: Reports, 49,
opulations of Dalbergia ecastaphyllum (L.) Taub. Biosci. j.(Online), 1789-1798.
ıtic Sea; carbonate production on the microscale of individual segments. PeerJ, 11, e15061.

ly differentiated flowers. PeerJ, 8, e8571.
ophyceae) segments. Plos one, 13(10), e0206492.
f segments in Halimeda tuna (Bryopsidales, Ulvophyceae). Hydrobiologia, 849(11), 2581-2594.
Glechoma hederacea (Lamiaceae). AoB Plants, 13(3), plab013.

imitation and biogeography within the well-known desmid species Micrasterias fimbriata and M. rotata
y, 60(1), 90-103.
genus Micrasterias (Desmidiales, Streptophyta). PLoS One, 9(1), e86247.
ed genotypes as new tool of Argania spinosa L. Skeels breeding. Acta fytotechn zootechn, 23(3), 125-138.
ica dioica (Urticaceae). Botanical Journal of the Linnean Society, 195(4), 606-621.
orellales: Jungermanniopsida), a small lineage of early land plants today. Botanical journal of the Linnean
ometry and pigmentation in Mammillaria haageana (Cactaceae). BMC plant biology, 22(1), 1-14.
cal intermediacy, novel fragrances and asymmetric pollen flow. Plant Biology, 21(2), 206-215.
or genomic prediction, and genome-wide association study of sorghum seed morphology. PloS one, 14(11),
the Linnean Society, 130(2), 410-418.
of stress?. Ecological indicators, 97, 457-465.

ng shoot apical meristems of a wide range of herbaceous angiosperms. American Journal of Botany, 107(1), 20-

`Nepenthes rafflesiana jack. Plants, 9(11), 1603.
ral populations. Trees, 31, 1697-1710.
ıtion of pollination syndromes in Caiophora (Loasaceae, subfam. Loasoideae). Annals of botany, 117(5), 937-
tric flower shape variation in species-level adaptive diversification–Calceolaria polyrhiza (Calceolariaceae) as a
contributes to fluctuating asymmetry in plants: first empirical evidence. Journal of Evolutionary Biology, 31(2),
rtual3D based morphospace for Pelargonium. PeerJ, 8, e8823.
n Flanders (northern part of Belgium). Trees, 29, 1661-1672.
ıevis reveals putative random ecotype evolution. Plant Systematics and Evolution, 302, 219-229.
ıred genetic basis. PeerJ, 9, e10672.
Botanical Journal of the Linnean Society, 179(2), 335-348.

elationships among three sympatric species and their hybrids. International Journal of Plant Sciences, 173(8), 875-
us (Fagaceae). Plant Biosystems, 143(3), 575-587.
ı of traditional and geometric morphometric methods. Plant Biosystems, 143(3), 564-574.
ology of Iris pumila-comparison of a polluted city area and the unpolluted Deliblato Sands (nature reserve). Appl.
Iercurialis perennis: a geometric morphometric examination. Archives of Biological Sciences, 68(4), 705-713.
ly of a hybrid line between actinomorphic and zygomorphic flowers. Frontiers in Plant Science, 6, 724.
ic morphometrics in Theobroma cacao. Applications in Plant Sciences, 11(5), e11549.
traditional and geometric morphometric methods, a pilot study. Journal of Forestry Research, 33(6), 1751-1764.
ıgy, 36(2), 233-250.
hometrics Approach. Plants, 12(12), 2381.
